# P-1980. CNS Toxicity Profile of Metronidazole: A Review of Spontaneous Reports in the AERS System

**DOI:** 10.1093/ofid/ofaf695.2147

**Published:** 2026-01-11

**Authors:** Manu Mathew, Ashin Siby, Jose T John

**Affiliations:** Durdans Hospital, Colombo, Western Province, Sri Lanka; Durdans Hospital, Colombo, Western Province, Sri Lanka; Durdans Hospital, Colombo, Western Province, Sri Lanka

## Abstract

**Background:**

Metronidazole is a widely prescribed nitroimidazole antibiotic for anaerobic and protozoal infections. While generally considered safe, rare central nervous system (CNS) toxicities—including encephalopathy, seizures, and peripheral neuropathy—have been documented. This study aimed to analyze the CNS toxicity profile of metronidazole based on post-marketing surveillance data from the U.S. FDA Adverse Event Reporting System (FAERS).

**Figure:**
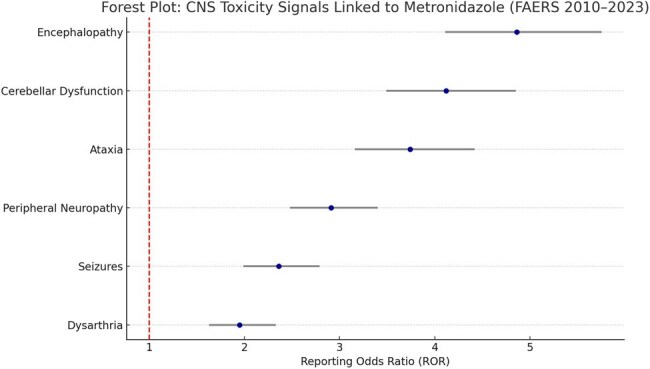
Forest Plot: CNS Toxicity Signals Linked to Metronidazole (FAERS 2010–2023) This forest plot presents Reporting Odds Ratios (RORs) with 95% confidence intervals for CNS adverse events associated with metronidazole, based on FAERS data. The strongest signals were observed for encephalopathy (ROR: 4.86), cerebellar dysfunction (ROR: 4.12), and ataxia (ROR: 3.74). These findings underscore the importance of monitoring neurological symptoms during prolonged therapy.

**Methods:**

A retrospective review of FAERS data from January 2010 to December 2023 was conducted. Reports identifying metronidazole as the primary suspect drug were screened for CNS-related Preferred Terms (PTs) using MedDRA, including encephalopathy, seizures, cerebellar dysfunction, dysarthria, ataxia, and peripheral neuropathy. Descriptive statistics were used to summarize event frequency. Disproportionality was assessed using Reporting Odds Ratios (RORs) with 95% confidence intervals (CIs), and a signal was defined as ROR lower CI >1 with ≥3 reports.

**Results:**

A total of 3,946 CNS-related adverse event reports were linked to metronidazole. The most frequently reported events included peripheral neuropathy (n = 1,423), encephalopathy (n = 978), ataxia (n = 516), and seizures (n = 441). The strongest signal was observed for metronidazole-associated encephalopathy (ROR: 4.86, 95% CI: 4.11–5.75), followed by cerebellar dysfunction (ROR: 4.12) and ataxia (ROR: 3.74). In 15.6% of reports, neurological symptoms persisted beyond discontinuation. Median time to onset was 10 days, with higher risk noted in patients receiving prolonged therapy ( >14 days).

**Conclusion:**

This FAERS-based analysis highlights a clear safety signal for CNS toxicity associated with metronidazole, especially in prolonged use. Clinicians should monitor for early neurological symptoms and consider timely discontinuation to prevent irreversible damage. Continued pharmacovigilance and patient education are critical to mitigating these risks.

**Disclosures:**

All Authors: No reported disclosures

